# NHS Activism: The Limits and Potentialities of a New Solidarity

**DOI:** 10.1080/01459740.2018.1532421

**Published:** 2018-11-14

**Authors:** Piyush Pushkar

**Affiliations:** Department of Social Anthropology, University of Manchester, Manchester, United Kingdom

**Keywords:** UK, consciousness, health care privatization, moral economy, NHS, solidarity

## Abstract

Using Thompson’s conceptualization of the moral economy, I describe how NHS activists in the UK utilize moral arguments to form alliances between different occupational groups, in a political battle against health care privatization, reflecting how a consciousness is being built upon solidarity and shared interests. In this context, professional duties of health care professionals are linked to the interests of all citizens. I explore how the deployment of professional ethics elides a moral hierarchy that may hinder the movement’s egalitarian potential.

The NHS, or National Health Service, is the UK’s state-funded health system. In this article, I investigate the moral arguments of people campaigning to “save the NHS” from funding cuts and privatization. I draw mainly on experiences in one English city of a campaign against mental health service cuts, and an industrial dispute involving junior doctors. I elucidate a moral logic that links the professional duty of health care professionals to improve the health of citizens to the interests of those same citizens. Since many health care professionals view health care privatization as a risk to the health of the nation, they join other activists in campaigning against it. I unpick this moral logic – a fusion of professional and civic ethics – and investigate how it is being mobilized, and so whether and how a consciousness is being built. By consciousness, I mean a perspective on and understanding of social structures and institutions, shared by a social group, on which actions are planned and executed to change and improve those structures and institutions for the benefit of that social group.

I conducted fieldwork with a group of activists of diverse backgrounds, but in the second half of this article, I focus on current and retired health care professionals. A return to Thompson’s (1991b [1971]) conceptualization of the moral economy can help understand the arguments of these activists as evidence of an alliance forming between workers of different occupations, who nonetheless identify with one another as having the same interests. Thompson’s formulation can also help us understand how activists choose particular aspects of past institutions to use as moral lenses through which to evaluate current socioeconomic relations and thus imagine the future. My interlocutors used the NHS as a symbol of the equal right of all to health care, but their deployment of professional ethics elides a moral hierarchy that may hinder the movement’s egalitarian potential.

In this article, I first outline Thompson’s usage of the concept of moral economy, and explain how I intend to use it. I then discuss existing anthropological work on health care privatization and what this article adds, and summarize the reforms that are taking place within the NHS. In then presenting my research, based on participant observation and interviews, I draw attention to the limits that may inhere within a consciousness shaped by professional ethics that favor the interests of one professional group.

## Thompson and the moral economy

Thompson (1991c) describes culture as being in continual flux, pointing not to shared understandings or “webs of significance” but to disagreements and protests. His point is not that consensus did not exist, but that it had to be fought for, with a fragile and contingent consciousness built around this struggle. The sides in the battles he describes are defined by class: patrician and plebeian, rich and poor, both groups negotiating large-scale structural and institutional changes as England industrialized and marketized.

Thompson sought to explain how and why food riots occurred at times of shortage in eighteenth century England. He objected to explanations of riots as “spasmodic” or “compulsive” (Thompson 1991b [1971]:185). Such mechanical explanations followed a simple formula: *if food is short, then riot*. Thompson instead concentrated on the understandings and agency of the “plebs”, a broad coalition of workers in different circumstances and on different incomes who came to see how they shared an identity of interests (Thompson 1980 [1963]). Artisans, miners, and shipbuilders saw that they were all affected by a shortage of affordable bread on the market (Thompson 1991b [1971]), and so they considered collective action to protect their interests. This may include other actions before, after or instead of rioting, choosing from a raft of possibilities including complaints, sanctions, shaming, and so on.

Thompson’s disavowal of mechanical explanations allowed a focus on how workers understood and voiced their experiences of exploitation and how this fed into their *justification* of their actions. In other words, his concern was not just their practice, but also their logic, or their “*mentalité*” (Thompson 1991b [1971]:260). He found that the marketization of the economy led to changes in the relationships between patricians and plebs. Ties between these groups were broken as the “paternalist” model was dismantled in favor of “free markets.” Therefore, when the price of bread fluctuated, workers could not rely on the social protection from hunger that they had once expected and understood as morally justified, that is, the rich and the producers and sellers of corn and bread had an *obligation* to make sure poor people could afford to eat. Thus, the poor objected to changes in the economy in *moral* terms: “A consistent traditional view of social norms and obligations, of the proper economic functions of several parties within the community … taken together, can be said to constitute the moral economy of the poor. An outrage to these moral assumptions, quite as much as actual deprivation, was the usual occasion for direct action” (Thompson 1991b [1971]:188).

Thompson restores agency to this group of oppressed people through a finely grained analysis of the ways in which they used particular customs, inherited, and chosen from the past, as tools to both apprehend and protest the present. They formed their moral arguments around these customs. Therefore, “the moral economy is summoned into being in resistance to the economy of the ‘free market’…The rationalizations or ‘modernizations’ of the capitalist market offended against community norms and continually called into being a ‘moral’ antagonist’” (Thompson 1991c:340). This implies a political conflict in which moral arguments are pitted against economic ones.

I argue, however, that taking account of moral arguments on all sides could shed light on contemporary battles. This is because Thompson’s use of the word “moral” refers to an understanding of the common good that encompasses not just customary rights, but also utopian aspirations (Edelman 2015). These workers were imagining and attempting to create a better future. Analyzing the conflicts and inconsistencies between and within moral arguments can illuminate the radical potential of the movements using them. Thompson demonstrates how their actions often worked to achieve the *limited* goals of the crowd, such as a temporary reduction in the price of bread. Their practices fitted into a particular logic which he calls “customary consciousness” (Thompson 1991a:1). The moral argument that constitutes this consciousness points toward its possible limits: *I know that the world-as-it-is is not as-it-ought-to-be, because it fails to live up to how-it-was.*

There is a tension between the limits and the potentialities of customary consciousness. Did people just want cheaper bread, or did they want to reform the world, make it fairer and more egalitarian? Thompson demonstrates that these two goals are not mutually exclusive. Put otherwise, the limits of customary consciousness were not binding for the radical imagination, and may even have contributed to its advancement. In earlier work (1980 [1963]), he described how class consciousness had developed in England, and evolved into larger-scale egalitarian movements such as the Chartists. This evolution was neither determined by shared interests nor shared experiences. However, “collective memories of struggles for a better world” (Schmidt 2015:30) were important to this developing consciousness and the working class’s armory of possible responses to oppression. What is important, then, to an analysis of a developing consciousness, is careful consideration of *which* aspects from *which past* feed into an imagined future. As well, how do people use these memories to forge links and alliances with particular others, and who do they consider class compatriots and who class enemies? How do moral arguments build on particular understandings of the past to undergird solidarity between workers of different backgrounds, and what limits might these understandings place on the imagination of the future? To contextualize my argument, I turn now to how morality has been considered in the anthropological literature on health care privatization.

## Morality and health care privatization

Privatization is defined by Krachler and Greer as “a change in ownership in which non-state actors become increasingly involved in provision, usually through a transfer of assets (e.g. the sale of a hospital) or an increase in work contracted out” (Krachler and Greer 2015:216). Much anthropological work on privatization in health care has taken place in low and middle income (Pfeiffer and Chapman 2010) and post-socialist (Stan 2015) countries. A significant body of work also exists in the USA (Dao and Mulligan 2016; Horton et al. 2014), where a fully comprehensive social insurance system has never existed. There has been much less ethnographic work in the UK. However, there have been many studies across disciplines on New Public Management (henceforth NPM) in the UK (Ferlie et al. 1996; Strathern 2000b). As discussed presently, NPM has often resulted in the privatization of public services, including health care.

The ethnographic literature documents the consequences of health care privatization for local populations, and their responses to it. Anthropologists have often described these consequences in morally weighted terms – in relation to oppression, immiseration, and inequality (Basilico et al. 2013; Pfeiffer and Chapman 2010). Drawing on Farmer’s work (1997, 2005), I suggest that the morality evinced by such terms is a judgement of the anthropologist rather than a focus of ethnographic investigation. Where morality is examined, it is often individual rather than group morality. This focus belies a relative paucity of literature on *collective* action against health care privatization. I situate the present study in this gap in the literature.

In poorer countries, especially in Latin America and Africa, anthropologists have examined the effects of structural adjustment programs (Basilico et al. 2013:87). Transnational organizations such as the World Bank and the International Monetary Fund encouraged many of these countries to reduce the provision of state services and to replace these by privatization. Health care became a commodity to be provided by the market rather than a right to be protected by the state. The World Bank argued that this would eventually lead to more efficiently delivered services with better access for citizens. Anthropologists who have investigated the actual experiences of individuals attempting to access these services have found increased immiseration, greater health inequalities (Pfeiffer and Chapman 2010) and worse public health indicators (Basilico et al. 2013). Farmer has elaborated these experiences, using the concept of structural violence (Farmer 1997) to describe and analyze the structural factors that lead to the suffering of the oppressed. He characterized the implementation of structural adjustment (and thus health care privatization) as one component of structural violence and, further, as a war on the poor (Farmer 2005). His work is largely focused on Haiti, where a strong, state-funded health system has never existed, but also on post-socialist countries such as Russia (Farmer 2005), where a previously existing state-funded health care infrastructure was dismantled, creating a space for privatized health care. Farmer finds less space for investigating how these groups might coalesce to take collective action to resist privatization.

One counter example is his description of the Zapatista rebellion (Farmer 2005), and the inclusion of the privatization of health care and subsequent restriction of access to it as grievances. In supporting the Zapatista cause, and imploring the reader to do so, he takes an explicitly moral stance in the evaluation of health care generally and privatization specifically. He argues that the duty of the health care professional, and the anthropologist, is to “bear witness” (Farmer 2005:27), to listen to the poor, since abuses of human rights are best understood from the perspective of those whose rights are being trampled. Solidarity begins with this attempt to identify with the people using health care (see also Horton et al. 2014 in the US). When Farmer describes the resistance of health care users, his emphasis is not on theorizing their morality, but affirming it. I emphasize this because he thus misses an opportunity to consider how the moral arguments of actors can contribute to the strengths and weaknesses of a social movement.

Scholars within organizational and management theory have discussed collective action within the context of NPM (Bezes et al. 2012), the logic of which bears some similarities with that of structural adjustment. Hood and Dixon define NPM as a “a set of loosely related ideas about government and public service reform… ostensibly intended to create ‘a government that works better and costs less’” (Hood and Dixon 2015:265). NPM seeks to produce more effective and efficient public services by way of combining top-down reorganization and surveillance with organizational downsizing and decentralization (Ferlie et al. 1996). Since it has often resulted in the privatization of public services, NPM has been described as a “market based ideology” (Ferlie et al. 1996:9). As this implies, managers are tasked with reorganizing and surveilling, and the group under surveillance include the broad and heterogeneous group of professionals that provide the services themselves, including health care professionals.

Literature within organizational theory (Bezes et al. 2012) thus pits these groups against each other, with professionals taking collective action not against marketization or privatization, but to protect their own professional autonomy. Bezes and colleagues also problematize the over-simplified dichotomy of professional versus bureaucrat, since different professional groups have different areas of expertise, different levels of professional prestige and “moral authority” (2012:9), and ultimately, different interests. Thus, professional collective action in the context of NPM often results in one professional group considering its interests narrowly, isolating itself and acting against other professional groups. They give several examples of French doctors taking actions to protect the autonomy only of the doctors working in one particular specialty.

Anthropologists have understood NPM as an aspect of audit culture, in which finance and ethics meet via the “the twin passage points of economic efficiency and good practice” (Strathern 2000a:1). Strathern stresses that the relationship between ethics and finance is not straight forward. Actors can use ethics to justify or resist audit. This raises the questions not just of what ethics are, but how they are used, to whose benefit. This is a question of politics.

Asking these political questions of how ethics are being used while considering Thompson’s reflections on consciousness will allow me to track not just lines of conflict, but also alliances. In contrast to the French doctors, Thompson was interested in how solidarity formed within and between groups, thus developing a consciousness of shared interests. Accordingly, I intend to focus on the political gains and losses that activists achieve through a consciousness of and focus on the shared interests of coalitions that span classes or occupational groups.

Bear and Mathur (2015) have written of the potential for shedding light on how the public good is imagined that may come from studying bureaucracies attempting to enact some of the same principles as NPM, such as marketization, fiscal austerity, and decentralization. They are not satisfied with a one-sided critique of bureaucrats as naked facilitators of neoliberal exploitation. Instead, they seek a more nuanced understanding of how new lines of conflict are opened up as individualized goals and techniques interact with visions of the public good that, insofar as they refer to the *public*, are inherently collective. I seek to follow their lead and combine it with Strathern’s analysis, to ask how a better future is being imagined, and for whom, as groups consider their interests narrowly or broadly. What orienting values are encapsulated in moral arguments, and how are they used, to serve whose interests? I now turn to the historical and political context in which NHS activists have been campaigning.

## Privatization and health care reform in the UK

The NHS is the UK’s public health care system, free at the point of delivery and paid for by general taxation.^1^ It has existed since 1948, when an already existing network of independent, charity, and military hospitals were combined under state control. It has been described as a “fundamental component of social solidarity and equal citizenship for over 60 years” (Leys and Player 2011:ix).

As a result of the NHS’s existence, private health care has not had much hold in the UK since 1948 (The King’s Fund 2015). A market was introduced in 1990, in which local health authorities “purchased” health care from hospitals and other “providers” of care. This was called an “internal” market because the providers all remained a part of the NHS and the funding was still provided by the government. When the Labour Party came to power in 1997, funding increased but the marketization process continued. Health authorities became Primary Care Trusts (PCTs), which were to commission services from providers of care, which were no longer limited to the NHS. As a result, the government created an “entry point” (Krachler and Greer 2015:215) for private health care, to provide care that would be commissioned and paid for by the state, remaining free to the patient. Although the introduction of commissioning was the “government’s main lever for opening the market to the private sector” (Krachler and Greer 2015:217), this aim was not declared. Instead, commissioning was justified on the basis of improving efficiency.

Limits on purchasing and borrowing remained until the Tory Party came to power and introduced the Health and Social Care Act (HaSCA) in 2012, under which PCTs were replaced by Clinical Commissioning Groups (CCGs), which were now able to purchase services from “any qualified provider.” This was “supposed to complete the project of making the NHS more efficient by fragmenting it into a system of independent trusts and subjecting them to market competition” (Leys 2016:12). The ostensible economic rationale was the same as with structural adjustment programs: exposure to competition with new private providers would cause state services to reduce cost *and* improve quality of care. Thus, marketization would supposedly benefit both patients and the state budget.

These reforms worried health care activists, not least because it was “a reorganisation so big you can see it from outer space” (David Nicholson, quoted in Cameron 2014) that came shortly after Prime Minister David Cameron’s manifesto promise of no top-down reorganizations. The message in the eyes of activists was clear: the government could not be trusted on the NHS. Campaigners examined the Act and came to their own conclusions, contra government rhetoric: that it would lead to fragmentation and duplication of services, that an inflated bureaucracy would increase cost, and that private providers would “cherry pick” easy, simple services while neglecting complex but essential services, leaving non-profitable gaps to be filled by state services (Pushkar 2012).

Although many of the people raising objections were doctors, the trade union that represents doctors in the UK, the British Medical Association (BMA), was criticized by its own members for not rejecting the HaSCA until very late. As well, the Royal Colleges, professional associations that represent individual specialties, were divided on whether to support or reject the HaSCA. Just as not all food shortages led to riots for Thompson’s research subjects, in this case, many doctors objected but the main bodies that represent them did not unite to pursue collective action.

At the time of writing, in early 2018, the Act had only achieved limited success in its unstated goal of introducing private providers into the health care infrastructure, and activists continue to decry that the Act has been a failure. There are declining levels of satisfaction, declining quality, increasing waiting times, increasing cancellations of procedures, reduced staffing levels, and a collapse of staff morale (Murray et al. 2016). Many services within trusts have been outsourced, such as cleaning, catering, and even some clinical services such as radiology reporting. NHS spending on independent sector care providers has steadily increased from 5.5% of the total spend in 2012/2013 to 7.6% in 2015/2016 (O’Leary 2017). However, very few full-service private hospitals have emerged (Leys 2016). When a private-equity controlled company, Circle, took over management of an already-existing NHS hospital in Cambridgeshire in 2012, its deficit doubled and care quality fell such that the UK’s monitoring organization, the Care Quality Commission, placed it under special measures. Circle walked away in 2015 and the NHS was left to pick up the pieces (Leys 2016).

Krachler and Greer (2015) suggest that a possible reason for this failure of privatization to take hold in the UK was a lack of money. In 2014, Simon Stevens, the chief executive of the NHS, instructed trusts to make £22 billion of savings by 2021–2022, and overall funding as a proportion of GDP is continuing to fall. Each local area has had to publish a Sustainability and Transformation Plan (STP), showing how it would make these efficiency savings, with emergency central funding only to be made available if sufficient savings were demonstrated (on how STPs operate, see Hammond et al. 2017). STPs do represent a change of direction from the HaSCA. The Department of Health’s emphasis is now on integration and partnership between health care organizations, as well as between health care and social care, rather than marketization and competition. However, since there has been no change in national legislation, CCGs remain compelled by the HaSCA to put new contracts out to tender. This ongoing compulsion, and ongoing fiscal austerity, has led most of the activists with whom I have spoken to see STPs as merely a “cover” for further cuts and privatization.

However, decreased funding of state health care in other countries, such as Germany, has created a space in which private health care has flourished (Krachler and Greer 2015). Why not in the UK? (Leys 2016) argues that one key reason has been political pressure from below. That is, campaigners’ efforts to keep health “highly politicized” (Krachler and Greer 2015:222) have contributed to the failure of private companies to gain a foothold in the UK. The efficiency logic of competition relies on supposedly better services outcompeting worse ones, leading to the closure of the latter. But the closure of hospitals leads to unacceptable gaps in patient care, which are anticipated by the activists who have been able to galvanize the public into campaigns to save several hospitals or specific services, most notably – and successfully – Lewisham Hospital in 2013. The closure of hospitals is therefore seen as “political suicide” for local politicians. The result is that these campaigns, by saving local hospitals, effectively stop a gap from being created that might have been filled by private health care.

## Methods

In 2012, well before starting anthropological research, I became involved in activism, initially campaigning against the imposition of the HaSCA. Since then, while continuing to work as a doctor in the NHS, I remained engaged in campaigns against reforms. At the time of the strikes I will describe, I was a BMA union rep in a major hospital in Manchester, England; I helped organize pickets, I spoke to many other junior doctors and other staff within the hospital, I collected signatures from members of the public on petitions, and I spoke to members of other unions. More recently, I suspended clinical work in order to conduct ethnographic fieldwork, with participant observation with other activists in the same city. Intermittent periods of fieldwork began in October 2016, followed by a sustained period from July 2017, due to finish in August 2018.

I set out to find people participating in any kind of political campaigns that mention the NHS. My only exclusion criterion was patient groups that campaigned on the basis of being patients, as my university ethical approval does not cover these groups. I spent most of my time with activists from three organizations: doctors in the BMA, activists affiliated to Keep Our NHS Public (KONP), and members of the Socialist Health Association (SHA). All three are national organizations with local branches, although the local branch of the SHA was inactive for several years until it was reconstituted in March 2018. In this article, I mostly discuss events in 2016, focusing on the BMA and KONP.

I attended organizing meetings, conferences and protests, sat at stalls in town centers and outside major events and handed out leaflets. I have also conducted 26 semi-structured interviews, and had informal discussions with other activists and health care professionals. Finally, communication within groups and with the wider public is very important to activists, and therefore, I non-systematically collated a large collection of organizing materials including posters, leaflets, emails and social media entries, starting from 2015. I have been active on Facebook, Twitter (@DrPiyushPushkar), Instagram (@DrPiyushPushkar), and Pinterest (@doctormagiot). While collecting this data, I continued to survey and reflect on it in my fieldnotes. These reflections have allowed me to use these materials as the basis for discussions with activists, and so social media entries were a substrate for further investigation rather than being taken at face value. Where I mention them below, I have chosen them as exemplifying some aspect of my argument that has been further corroborated by interviews and participant observation.

## The moral economy of NHS activism

In the city where I was conducting fieldwork, a drive to streamline services led not to closures of whole hospitals, but to “downgrading,” i.e. a reduction of services provided. This meant that either a service was lost completely, or people would have to travel further to another hospital to avail of the service. The former was the case with proposed mental health service cuts, including recovery, specialist psychological and dual diagnosis services for those who suffer with both mental health problems and substance abuse disorders.

I focus on three issues from the campaigns against these cuts that illustrate broader points that can be generalized to all of the campaigns I observed. Cuts to specific services become *local* sites of *national* resistance. By this, I mean that activists frame their campaigns not just as an effort to save that local service, but also to “Save the NHS.” The campaign against mental health service cuts had a tagline “a national scandal, a local crisis.” This discourse is aided by national campaigning groups that have local chapters, such as Keep Our NHS Public. The effect of this tactic is to broaden the “community of fate” (Levi and Olsen 2000:312). A cut to a service that affects people with a particular type of illness, in a particular place, is reinterpreted as an assault on the body that runs all health care services nationally, thus allowing activists to argue that all citizens are affected.

There is a moral tenor to the language of these campaigns. Words such as “scandal” point to how the reforms are understood as morally *wrong*. Words such as “threatened” (see below) and “save” point to the urgency of the issue.

For these campaigners, closures, cuts, and privatization are seen as of a piece. An open letter from campaigners read: “The clinics provided by the dual diagnosis service, which is a specialist service for people who have mental health and addictions, are threatened with closure. The Brian Hore Unit, the only specialist unit in Manchester, is also due to close, as part of the privatisation of alcohol and drugs services.”

In the minds of activists, cuts, closures, and privatization combine as one and the same thing. This is not due to fuzzy-headedness or hysteria, but rather, recognition of the inter-connectedness of these different processes. As one activist, Denise,^2^ wrote on her Facebook wall: “It’s all part of the plan.” One much-shared meme for NHS activists on social media is a Noam Chomsky quotation: “Defund, make sure things don’t work, people get angry and you hand it over to private capital.” In conversation, activists emphasized that the links between cuts and privatization do not play out by accident, but by design, i.e. cuts are made deliberately to bring about privatization, which is seen as the end goal. Campaigners see this end goal of privatization as morally wrong, more so for being deliberate. Another social media post from January 2017, in reference to doctors leaving the UK, explains how cuts lead to privatization, again invoking an urgency to the situation:

Another example of staff leaving. The aim is to create NHS failure. They do this by privatising, cutting, deliberately antagonizing staff, and rationing. Each feeds off the others to create a perfect storm of #NHSCrisis. We need the NHS Bill. We need mass opposition (March 4^th^ demo!) and we need trade unions to do something. We face losing our NHS otherwise.

This last quotation speaks to one of the solutions envisaged by the activists with whom I work: the NHS Reinstatement Bill. This parliamentary bill seeks to restore the NHS *as it was*. It does more than just revoke the HaSCA, and instead proposes to reverse the marketization process and abolish the purchaser-provider split, thus ending commissioning. I emphasize this point because it begins to suggest *what* activists see as wrong with the reforms. Frequently, they refer to its origins, to the politicians and principles associated with the NHS’s founding history, such as Aneurin Bevan, the Minister for Health from 1945–1951, who is either directly quoted or indirectly invoked. As one activist, Debbie, told me in an interview in November 2016: “Well, I think [the NHS] should provide what it always has provided, which is a universal health care service that’s state-provided and free at the point of access.” The point is that activists consider that the problem with the current reforms is that they are creating a situation in which the NHS will not be able to do *what it used to do*. A service, experienced in the past and understood as that which the state should provide, is perceived as being removed, an obligation not met, a promise broken.

Like Thompson’s plebs, NHS activists apprehend and protest the present in moral terms, seeking to reproduce the structures and institutions of the past. Can this be understood as a conservative tendency? For Thompson, the analogous question was: Did they want to radically restructure society, or did they just want more bread? Graeber noted the same issue among activists in New York City before Occupy, and among UK Uncut activists in England (Graeber 2013). They were also campaigning against government service cuts, and Graeber breathlessly asks: “How did we get to a point where the *radical* position is to keep things exactly the way they are? … If anything the message was reactionary: *stop the cuts!* What, and go back to the lost paradise of 2009? Or even 1959, or 1979?” (2013:20). For Thompson, it was important to focus on *which* aspects of *which* past were being reached for. Why were people of diverse occupations referring to the past, and, importantly, what kinds of alliances were being built in the process? I seek to answer similar questions with NHS activists.

First, who are these activists? They describe themselves as engaged citizens, angry patients and concerned health care professionals or ex-health care professionals. The current and retired health care professionals form an important and trusted contingent among activists, and in this article I focus on them. I note an alignment between professional and civic duties that feeds into their moral logic. These professionals’ knowledge of and facility with the internal machinations of the health system and the discourses around it mean that they are able to parse governmental rhetoric and documents that remain opaque to many people. These dense documents require hours of painstaking perusal, but these activists see it as a key part of their role to fully understand their arguments, test their assumptions, check their facts and figures, and challenge decision-makers when they find errors or inconsistencies.

However, finding mistakes is not the only goal. Another key motivation is to understand how the reforms laid out will lead to privatization. As Debbie, a retired social worker, told me,

I think the government has not been honest about what they are doing. There’s such a lack of transparency. It’s mind-boggling that activists like us have to do as I’ve told you, sit at home reading pages and pages of Sustainability and Transformation Plans to find out *what’s actually going on*. There are lots of lies being told about what funding is being given and how it’s being used. And, basically, I object because it looks to me as if services are going to be cut, withdrawn, charges are going to be imposed. Everything that I think is wrong in terms of health policy, and that I think the majority of the public would disagree with as well if they knew what was going on. And I guess one of the worst things is, well who am I to say, if the majority of the country wants a privatized health service, that’s their opinion. But if they’re given the facts and figures and information to decide on these things, that’s the way it should go. And on top of everything there’s a lot of language being used that’s disingenuous, that doesn’t say what it means. On top of it, it feels like double-speak.

Activists peruse available documents, attend council committees and NHS conferences, arrange meetings with managers and politicians, collecting information to answer the following question: how will these reforms lead to privatization? The above quotation illustrates several stages in the moral and practical logic of NHS activism:

1. *What’s actually going on?* – the government is seeking to defund the NHS in order to create a space for privatization.
2. This will lead to a worse service for those unable to afford the best private health care. Such people will inevitably suffer and be less healthy.
3. Therefore, cutting essential services and the privatization of health care are interconnected, and both morally wrong.
4. Government documents, plans, and declarations do not reflect *what’s actually going on*.
5. It is an engaged and caring citizen/health care professional’s *duty* to read through the documents in order to assess the likely effects for other patients and citizens and then educate them, i.e. “raise awareness”, through petitions, leaflets, books, and so on.

This alignment of professional and civic duties in the moral logic of activists politicizes them. In order to improve the health of all people, they must engage in the political process to campaign against privatization. Thus an alliance forms between health care worker and health care user. However, an alignment of civic and professional duties did not result in collective action to resist government reforms when the HaSCA was announced in 2012. Among doctors, there was much disagreement. The BMA and Royal Colleges did not unite in the outright rejection of the Act. Conversely, when the BMA balloted for strike action in 2015, 98% voted in favor. I now turn to this dispute to unpick the discrepancy between professional disagreement in 2012 and greater unity in resistance in 2015–16.

For most of 2016, junior doctors^3^ in England, represented by the BMA, were engaged in an industrial relations dispute with the government’s Department of Health. The conflict concerned a new contract that proposed to extend normal working hours to include evenings and weekends. There were four strikes, with each strike leading to further negotiations and an improvement in the contract offered. The pickets took place outside hospitals on strike days: 12 January, 10 February, 9–10 March and 26–27 April. Junior doctors were joined by other health care professionals, including nurses, physiotherapists, and social workers, other NHS staff including cleaners and administrative staff, and non-NHS staff including accountants, lecturers and, teachers. Local café workers brought fruit, cake, and hot drinks.

I do not wish to dwell on the intricacies of the contract offer itself, but focus on two points regarding how doctors and NHS activists behaved on the pickets. First, doctors emphasized their moral duty to strike. Morally weighted chants reverberated: “Not safe! Not fair! Not safe! Not fair!” Doctors stated that it was their moral duty to provide safe care, and referred to the professional “duties of a doctor” described by the (General Medical Council 2013), the body that regulates doctors in the UK. Providing safe care was widely deemed impossible or, at least, less likely, under the new contract, thus doctors felt justified in striking. The short term inconvenience for some patients would be outweighed by the potential harm that might come to other patients in the long term if the contract were to be imposed. Protestors repeatedly emphasized on the pickets and in the media that the strikes were not to do with pay, but with patient safety.

Other chants sought to shame the Secretary of State for Health, Jeremy Hunt, who was judged to be in hiding: “Where are you Jeremy? Where are you Jeremy?” Hunt, of course, was not present at the pickets. The chant’s question was rhetorical, addressed to the people who were present – doctors and NHS activists – and was a *tactic* in a moral battle against a government that was also presenting its own moral arguments: Hunt claimed that the contract enabled the government to stick to one of its manifesto promises, bringing in a “7 day NHS.” He cited evidence (Freemantle et al. 2015) of a “weekend effect” such that more people die in hospital at weekends than during weekdays. Thus he was able to claim that his own actions were also part of a quest for patient safety.^4^

Describing a moral argument as a “tactic” and the industrial dispute as a “battle” brings into view not just what the moral arguments are, but also how they are *deployed*, by whom, and to what ends. Shaming Hunt pits him as the enemy. However, recognition of a common enemy is understood by social movement scholars as a necessary but not sufficient condition for generating collective action (Levi and Olsen 2000). Levi and Olsen argue that common interests and a common program are also required to achieve victories. They describe three kinds of community of interest, each broader and more difficult to maintain than the last: interests shared by those of the same occupation, interests shared by members of a single organization or industry, and universal interests. As universalistic interests come into conflict with particularistic claims, social movements must always look for ways to maintain solidarity in order to preserve longevity.

Another common chant illustrated how activists and doctors were beginning to understand that they had interests in common: “Save our NHS! Save our NHS!” Although the contract issue bore no direct relation to cuts of specific services, or of privatization, it was still interpreted as some kind of attack on the NHS, that is, the provider of universal health services for UK citizens. The groups present at pickets, consisting of doctors, NHS activists and other workers, appeared to be building a recognition that they were all members of one social group from whom something was being taken away by the common enemy: those in control of dispensing the state’s obligations.

To summarize, when a narrow community of interest – doctors, united by occupation – was threatened, they found a way to build solidarity with other activists and the wider public by broadening the community of fate, by arguing that the entire NHS was at stake. The peculiarity of using the NHS to broaden the community of fate is that it turns the first kind of community of interest described by (Levi and Olsen 2000) into *both* the second and the third types simultaneously, since the NHS is understood as a universal service available to all citizens.

Thus the “marketplace bargaining power” (Silver 2003:13) afforded to doctors by their years of highly specialized training and limited numbers was augmented by associating their industrial dispute with the survival of the NHS. The obverse of this was that NHS activists stood to gain from the perceived moral and professional authority of doctors. That is, activists who had experienced an “orientalization” (Theodossopoulos 2014:488) of their arguments as “emotional” or “misinformed” when raising their objections with managers and politicians, could now counter such dismissals by linking them with an “authoritative story” (Biehl 2013:412) told by doctors. Indeed, at the time of the strikes, both doctors and activists spoke a great deal about the fact that the public trusted doctors more than politicians.

Activists and doctors recognized a “mutuality of interests” (Guha 1999:27) on the pickets, which I read as evidence of the building of a conjoined consciousness. I do not wish to overstate the maturity of this consciousness, by which I mean the level of consensus among the public, or even among NHS activists and doctors. Hunt’s contract was eventually imposed, leading to acrimony and disunity not just between professions, but within the BMA itself. However, I have found that among the activists with whom I have been working, one key aspect of their arguments focuses on recognizing a mutuality of interests among all patients of the NHS, with an emphasis on defining this as broadly and equally as possible. The professional duty to maintain the health of the nation fed into the understanding of shared interests. In recognizing shared interests, health care professionals and NHS activists appear to work with an understanding of class relations that sees both groups combining with other users of the NHS as a coalition. The fight is against those who seek to undermine equality by defunding and privatizing health care. The point is not that consensus exists, but that activists are fighting to build such a consensus to consolidate solidarity among workers of different occupations and backgrounds.

## Professional ethics and customary consciousness

What limits or vulnerabilities are there in the kind of consciousness I have described? I have argued that activists benefited from the “authoritative story” (Biehl 2013:412) told by doctors. One source of the perceived authority of professions is their ethical codes (Pels 2000). In the case of doctors, that is *Good Medical Practice* (General Medical Council 2013), the same code used here to justify collective action. In the UK, doctors must be registered with the GMC in order to practice, and are obligated to follow its ethical code. If found to be in breach, doctors can be penalized and the GMC can revoke their licence to practice. Pels argues that ethical codes guard the “competence and honour of the professional” (Pels 2000:139) in the eyes of the public. That is, professional ethics elevate the professional in the social hierarchy.

Pels adds that such codes are often used as a “prophylactic” (Pels 2000:155) to isolate the practice of professional ethics from politics. In contrast, in this dispute doctors deployed their professional ethics in such a way as to maximize their full political impact, which was to extend solidarity as widely as possible. Doctors used the duty to protect patient safety to justify collective action alongside non-medical NHS activists. Here we see the vulnerability of the kind of consciousness being built, as professional ethics also signify the separation of doctors. The very ethical code that is being used as part of a moral argument to build a single community of fate is the same ethical code that separates medicine as a “vocation”, signifying a perceived moral authority over other lines of work.

Thompson’s understanding of shared consciousness between different occupational groups can help shed light on this inconsistency. For Thompson, economic and structural changes led to changes in class relations that people protested in moral terms. The protestors based their moral arguments on customs learnt from the feudal, deeply unequal past. They built a consciousness that recognized solidarity across different occupational groups, but with inherent inconsistencies and subsequent buffers placed on the radical imagination. As already noted, there was a tension between whether they were aiming for cheaper bread, or for a radically egalitarian future.

Junior doctors and NHS activists are building a consciousness that recognizes solidarity across class lines. However, I have explained how their moral argument introduces a vulnerability within that solidarity, since the ethical code itself posits a hierarchy within which professionals are held in higher esteem. Similarly, there was a tension among doctors in the industrial dispute. Not all doctors understood themselves as engaged in a battle to “save the NHS”. The analogue of the tension between cheaper bread and a radically egalitarian future was the tension I noted on the pickets between simply wanting a better contract, and wanting an NHS that was better for all. Of course, as explained, it was in doctors’ interests to elide these two demands, which they were able to do using the GMC’s ethical code, a moral argument learnt in an unequal present.

For this coalitional consciousness to evolve to imagining a more egalitarian future, it will have to capitalize on the solidarity formed in this struggle, while forging a new moral reasoning that is not based on inherent inequality. Whether this is likely to happen or not will depend on whether doctors conceive of their interests narrowly, in separation from other social groups, or broadly, in coalition with other groups. I have argued not that there is a consensus that doctors are members of a single community of interest with other UK citizens, but that some doctors and activists are fighting to build a consciousness that recognizes such a community of fate.

## Conclusion

In 2012, there was limited collective action from the BMA and medical professional associations to resist the HaSCA. In 2016, when doctors’ own interests were threatened by a new contract, the profession showed more unity in its resistance, with 98% of junior doctors voting to strike. At the pickets, they used moral arguments not only to emphasize the threat to their own interests but to emphasize the effects on patient safety and the NHS itself.

Rather than conclude that doctors were using the broader arguments opportunistically, *only* to further advance their own interests, I have unpicked the consequences of this interlinking of professional and civic duties. Using the concept of consciousness has brought into view how a dispute rooted in professional self-interest can contribute to wider solidarity, building coalitions across different classes. However, I have speculated that for the consciousness to evolve further into being able to imagine a radically egalitarian future, doctors and NHS activists will have to develop new moral arguments that are not tied to the institutions that perpetuate an unequal present.

## Notes

1. There is a flat rate prescription charge with some exemptions, e.g. children. For dentistry and eye care, free-of-charge services are very limited.

2. All names have been changed to protect anonymity.

3. In the UK, a doctor is a “junior doctor” from the point of finishing medical school, until they finish postgraduate training, i.e. all doctors are junior doctors until they become consultant specialists or general practitioners.

4. Hunt’s analysis of the evidence was contested by doctors (Rimmer and Kmietowicz 2015), opening a technical-scientific aspect to the dispute. In this article, I focus on the moral aspect.
